# Hyporegenerative anemia and other complications of rhesus hemolytic disease: to treat or not to treat is the question

**DOI:** 10.11604/pamj.2019.32.120.17757

**Published:** 2019-03-14

**Authors:** Aqeel Abdullah Alaqeel

**Affiliations:** 1 Pediatrics, College of Medicine, Qassim University, Qassim, Saudi Arabia

**Keywords:** Rhesus haemolytic disease, hyporegenerative anaemia, erythropoietin

## Abstract

Rhesus hemolytic disease of the newborn is rarely found after the implementation of anti-D immunoglobulin prophylaxis. However, it may lead to cholestasis, elevated liver transaminases, iron overload and late hyporegenerative anemia when it occurs. Etiology of this type of anemia is not defined yet and treatment is controversial. It is typically recognized after two weeks of life which is characterized by low hemoglobin and reticulocyte count. We have reported a case of a neonate with Rh hemolytic disease with late hyporegenerative anemia that was noted at day 18 of life. We treated this anemia by erythropoietin (EPO) 250 U/kg three times per week. Two weeks after initiation of erythropoietin treatment, a stable hemoglobin was noted along with an increased reticulocyte count. The patient required one further blood transfusion in the third week of therapy. Other associated findings were self-limited. A year of follow-up showed an appropriate development for age.

## Introduction

Rh(D) alloimmunization occurs when an Rh(D)-negative mother develops antibodies against Rh(D) antigen. Alloimmunization can lead to hemolytic disease of an Rh(D)-positive newborn and/or fetus. Maternal IgG Anti-D antibodies cross the placenta and destroy fetal red blood cells. RhD incompatibility can be either asymptomatic with mild anemia or present with severe anemia which leads to heart failure and subsequent hydrops fetalis or stillbirth [1, 2]. Despite the worldwide use of anti-D immunoglobulin prophylaxis, there are several reported cases of women with Rh alloimmunization and (0.04%) newborns with rhesus hemolytic disease (RHD) [3]. In the setting of prolonged hemolysis and repeated blood transfusions, cholestasis, iron overload and late hyporegenerative anemia have been reported [4-6]. Late hyporegenerative anemia is caused by depressed erythropoiesis and is characterized by low reticulocyte count. Management of this type of anemia by EPO is controversial [7], however, this case was managed by EPO and showed a successful response. We report a case with one year of follow-up of RHD complications.

## Patient and observation

**Figure 1:**
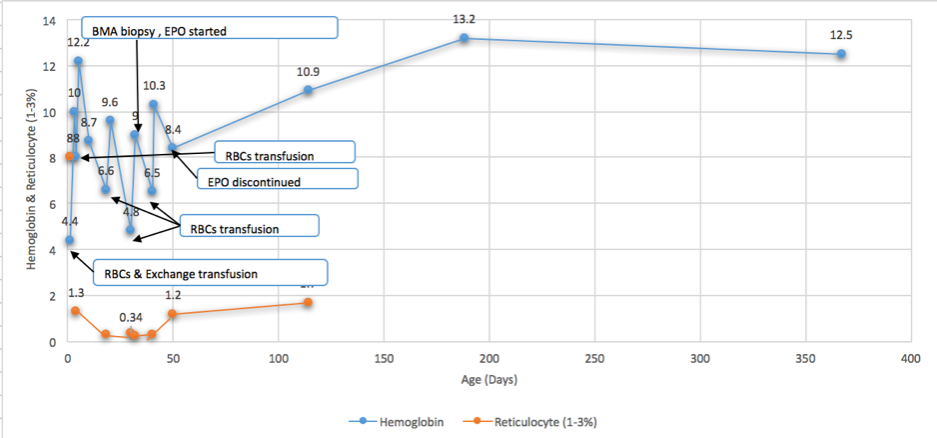
Hemoglobin and reticulocyte from birth until 1 year of age

**Figure 2:**
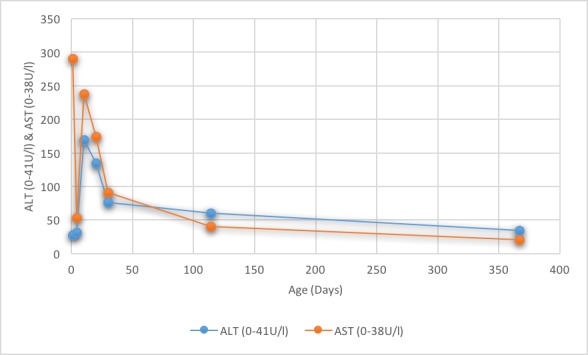
Liver enzymes (ALT and AST) from birth until 1 year of age

We present a case of a newborn boy who was delivered uneventfully weighing 3.5kg, after uncomplicated pregnancy to a 29-year-old women G2 P2 at 37 weeks of gestation. Apgar scores were eight and six at 1 and 5 minutes, respectively. Subsequently, the baby showed lethargy and decreased oxygen saturation for which, he was resuscitated and intubated then started on indomethacin for suspicion of duct dependent congenital heart disease. On initial examination, the baby was limp and severely pale with no apparent congenital anomalies, dysmorphic or hydropic features. Subsequently, the baby was transferred to the neonatal intensive care unit. Urgent echocardiography demonstrated mild-moderate pulmonary hypertension with no structural heart defects. Initial blood workup including a complete blood count at birth revealed hemoglobin: 4.4 g/dL, hematocrit: 14.30%, platelet: 78,000 K/μL, direct Coomb's test was markedly positive, elevated reticulocytes and O Rh-positive blood. The mother blood group and Rh is O negative with the previous baby of O positive. Thus, Rh incompatibility diagnosis was made and treatment was started. She had one healthy Rh-positive child from her first pregnancy. Of note, in the present pregnancy, an anti-D antibody was not tested and the fetus did not receive an intrauterine blood transfusion. The mother received Rho(D) immunoglobulin in the first 72-hr post her prior delivery and reportedly received another dose at 32 weeks of gestation of this pregnancy. Our patient received a blood transfusion and then an exchange transfusion. Furthermore, he had been stabilized with fluids and antibiotics. At day 3 of life, he was extubated, however, he continued to have decreased hemoglobin (Figure 1) with increased liver transaminases with a peak aspartate aminotransferase (AST) of 238 U/L (normal ranges, 0 to 38U/L) and alanine aminotransferase (ALT) of 169 U/L (normal ranges, 0 to 41 U/L) (Figure 2).

**Figure 3:**
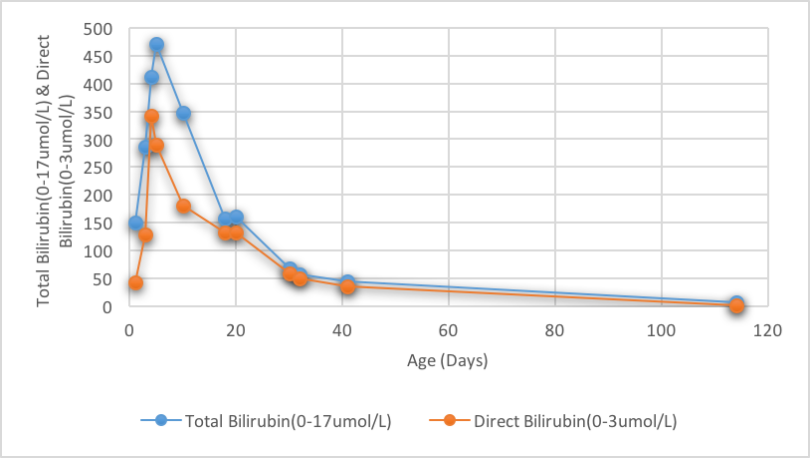
Total and direct bilirubin in the first 4 months of life

**Figure 4:**
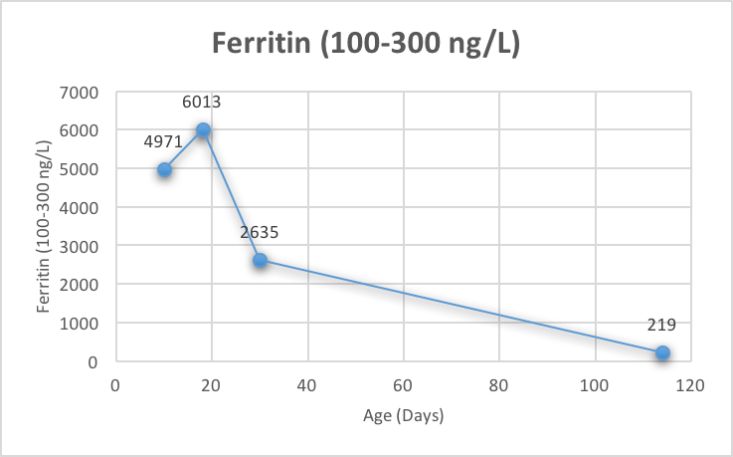
Ferritin level in the first 4 months of life

Also, total and direct bilirubin continue to increase and reached a peak of 472 umol/L (normal ranges, 0 to 17) and 343 umol/L (normal ranges, 0 to 3), respectively (Figure 3). Ursodeoxycholic acid was given as a treatment for cholestatic jaundice for a short period. Platelet count was low; 56,000 to 138,000 in the first week then normalized and continued to be normal without platelet transfusions. Reticulocyte count was initially elevated then noticed to be inappropriately low (0.29%) to the hemoglobin level upon discharge. Additionally, serum ferritin found to be elevated 6013 ng/L was attributed to repeated blood transfusions (Figure 4). The patient was discharged home at 20 days of life, with hemoglobin of 9.6 g/dL, after receiving a total of three blood transfusions with a plan of weekly follow-up. At 30 days of age, he was hospitalized for anemia with a hemoglobin of 4.8 g/dL and reticulocyte count of 0.34%. Total and conjugated serum bilirubin and ferritin levels were elevated, however; they were lower than prior levels. On physical examination, he was icteric and tachycardic with no hepatosplenomegaly. Therefore, he received packed red cell transfusion. With persistent low hemoglobin and reticulocyte count, bone marrow biopsy was performed which showed normal cellularity. Hyporegenerative anemia diagnosis was made secondary to Rh incompatibility for which treatment with recombinant human erythropoietin (250 U/kg by subcutaneous injection, three times a week) was initiated on day 32 of life. Ten days later another transfusion was necessary (Hb was 6.5 g/dL and reticulocyte 0.3%). In 3 weeks of EPO treatment, reticulocyte count increased to 1.2% and hemoglobin level stabilized. Erythropoietin was discontinued at four weeks of treatment. Other causes of conjugated hyperbilirubinemia were excluded. Work-up was negative for TANDEM mass spectrometry, urine-blood amino acid, α1-antitrypsin, serological test results for TORCH, hepatitis A, B and C, parvovirus, and reducing substances. Additionally, endocrine work-up, coagulation profile and abdominal ultrasound were normal. On follow up, bilirubin (Figure 3) and ferritin (Figure 4) levels decreased and normalized at four months of age while liver markers normalized at one year (Figure 2). No chelation therapy was needed to normalize the ferritin level. The patient had been followed at 4, 6 and 12 months of age at which he was meeting normal developmental milestones for age.

## Discussion

Hyporegenerative anemia is characterized by depressed erythropoiesis and reticulocyte count. It is commonly seen between 2 to 6 weeks after birth and the pathogenesis is still unclear [8, 9]. Claire Nicaise *et al*. reported a successful treatment by EPO for two neonates with late hyporegenerative anemia; also, his review showed that the treatment by EPO decreased the need for erythrocyte transfusions [10]. Other authors have reported that treatment with EPO may be ineffective when anti-Rh(D) antibody titers are high despite a 5-week course therapy [5]. The optimal dose and time of initiating therapy have not been defined. We followed the protocol described by different authors for this indication: 250 U/kg three times a week [5, 10, 11]. Our patient showed the criteria of hyporegenerative anemia with reticulocytopenia that started after two weeks of age with a persistent requirement of blood transfusions. Fortunately, our patient showed a response to EPO in the form of stable hemoglobin without blood transfusion and an increase in reticulocyte count (Figure 1). A differential diagnoses of neonate with cholestasis include infections (sepsis, urinary tract infection, toxoplasmosis, rubella, cytomegalovirus, human herpesvirus 6, syphilis, parvovirus B19, hepatitis B and C), inborn errors of metabolism, including 1 -antitrypsin deficiency, galactosemia, cystic fibrosis, tyrosinemia and progressive familial intrahepatic cholestasis, endocrinopathies (hypothyroidism and hypopituitarism) and inspissated bile syndrome [12]. In our patient, in the light of Rh(D) alloimmunization with prolonged hemolysis and repeated blood transfusions, most likely inspissated bile syndrome is the cause secondary to iron overload, provided that other causes were ruled out. A study showed that five of 35 neonates with RHD (14%) had cholestasis and all those five neonates had iron overload at birth [13]. Smits-Wintjens VE *et al*. confirmed the association of cholestasis in infants with red cell alloimmune hemolytic disease in 13% (41/313). Furthermore, cholestasis resolved spontaneously within one week to 3 months after birth in almost half of the patients [14]. In our case, the infant had resolved cholestasis by 3-4 months of age (Figure 3) and liver function was slightly elevated at that age but normalized in a year (Figure 2). Iron overload is recognized and reported in neonates with red cell alloimmune hemolytic disease which likely due to prolonged hemolysis and repeated blood transfusions [4, 7]. A study on sixteen neonates with RHD showed that all had elevated ferritin irrespective of intrauterine transfusions [15]. Rath ME *et al*. reported that in neonates with red cell alloimmune hemolytic disease, an iron overload occurs in 70% of neonates at birth, 50% at the age of 1 month and 18% at the age of 3 months and incidence of iron overload gradually decreases within the first three months [13]. Several case reports have treated iron overload with a chelating agent (Desferrioxamine) however, ferritin levels were either robustly elevated or after finding an increased iron store in liver biopsy [4, 16]. Our patient showed elevated ferritin initially which is correlated with bilirubin level and liver transaminases, subsequently, it has decreased gradually with no interventions or chelation therapy and reached to a reasonable level once the blood transfusions discontinued (Figure 4).

## Conclusion

RHD continues to occur despite anti-D immunoglobulin prophylaxis, possible reasons are reported by Biffoni F *et al*. [17]. Cholestasis, elevated liver enzymes, iron overload and hyporegenerative anemia, are associated with RHD infants. Hyporegenerative anemia can be treated by EPO in order to decrease the blood transfusion requirement and eventually maintain normal hemoglobin level. Per our case and other reported cases, we recommend treating by EPO. However, more extensive studies are warranted to evaluate EPO therapy. Other complications are likely self-limited but need to be monitored closely. Our case is unique as it showed a whole year of follow-up and approach to different complications of RHD.

## Competing interests

The author declares no competing interest.
